# Sharing is caring? Measurement error and the issues arising from combining 3D morphometric datasets

**DOI:** 10.1002/ece3.3256

**Published:** 2017-07-31

**Authors:** Carmelo Fruciano, Mélina A. Celik, Kaylene Butler, Tom Dooley, Vera Weisbecker, Matthew J. Phillips

**Affiliations:** ^1^ School of Earth, Environmental and Biological Sciences Queensland University of Technology Brisbane Qld Australia; ^2^ School of Earth and Environmental Sciences University of Queensland St. Lucia Qld Australia; ^3^ School of Biological Sciences University of Queensland St. Lucia Qld Australia

**Keywords:** geometric morphometrics, measurement error, photogrammetry, phylogenetic signal

## Abstract

Geometric morphometrics is routinely used in ecology and evolution and morphometric datasets are increasingly shared among researchers, allowing for more comprehensive studies and higher statistical power (as a consequence of increased sample size). However, sharing of morphometric data opens up the question of how much nonbiologically relevant variation (i.e., measurement error) is introduced in the resulting datasets and how this variation affects analyses. We perform a set of analyses based on an empirical 3D geometric morphometric dataset. In particular, we quantify the amount of error associated with combining data from multiple devices and digitized by multiple operators and test for the presence of bias. We also extend these analyses to a dataset obtained with a recently developed automated method, which does not require human‐digitized landmarks. Further, we analyze how measurement error affects estimates of phylogenetic signal and how its effect compares with the effect of phylogenetic uncertainty. We show that measurement error can be substantial when combining surface models produced by different devices and even more among landmarks digitized by different operators. We also document the presence of small, but significant, amounts of nonrandom error (i.e., bias). Measurement error is heavily reduced by excluding landmarks that are difficult to digitize. The automated method we tested had low levels of error, if used in combination with a procedure for dimensionality reduction. Estimates of phylogenetic signal can be more affected by measurement error than by phylogenetic uncertainty. Our results generally highlight the importance of landmark choice and the usefulness of estimating measurement error. Further, measurement error may limit comparisons of estimates of phylogenetic signal across studies if these have been performed using different devices or by different operators. Finally, we also show how widely held assumptions do not always hold true, particularly that measurement error affects inference more at a shallower phylogenetic scale and that automated methods perform worse than human digitization.

## 1 INTRODUCTION

Geometric morphometrics has become the method of choice for quantitative morphological studies because it combines statistical rigor and ease of visualization and allows for a separation of shape and size (Adams, Rohlf, & Slice, 2004, 2013; Zelditch, Swiderski, & Sheets, 2004). For these reasons, geometric morphometric data are frequently generated for a wide range of organisms and their parts and to address a wide array of evolutionary questions. With increasing frequency, geometric morphometric datasets are also shared among researchers. Data are shared among researchers in the same laboratory and among researchers in different laboratories through private contact or public repositories. Data are increasingly shared through either specialized (Copes, Lucas, Thostenson, Hoekstra, & Boyer, 2016) or generic (e.g., Dryad, http://datadryad.org/) public repositories. Indeed, a search for “geometric morphometrics” in Dryad reveals a clear trend of increase in the number of deposited morphometric datasets (Fig. S1). Data are typically shared in the form of landmark coordinates or as the raw data on which landmarks are digitized—for example, pictures for 2D analyses and surface models for 3D analyses. The sharing of morphometric datasets has many advantages, including a potential increase in statistical power due to increased sample sizes and the ability to tackle broader questions with datasets which include more and more species. Indeed, it has recently been suggested that “crowdsourcing” the acquisition of geometric morphometric data is a viable option to reduce the time researchers spend acquiring data (Chang & Alfaro, 2016). However, sharing morphometric datasets also creates the situation in which data obtained from multiple devices and/or operators are combined. This, in turn, creates the risk that variation in the way data have been acquired distorts subsequent analyses (i.e., can potentially increase measurement error). Although no empirical investigation is free from measurement error, its extent and its effect on inference are largely unexplored in geometric morphometrics (Arnqvist & Mårtensson, 1998; Fruciano, 2016). In particular, random measurement error increases variance and is typically thought to confound biological patterns by decreasing the “signal‐to‐noise ratio” (Arnqvist & Mårtensson, 1998; Fruciano, 2016; Yezerinac, Lougheed, & Handford, 1992). A reasonable—but largely untested—consequence of this is that measurement error should affect analyses more seriously when biological signal is relatively weak. For instance, measurement error might be more serious in intraspecific, as opposed to interspecific data. Another issue is that nonrandom measurement error (i.e., bias) has the potential to affect the computation of means, so that differences induced by error are incorporated in the analysis as true differences between groups (Fruciano, 2016). Here, we investigate the magnitude of random measurement error introduced by combining 3D geometric morphometric data obtained with multiple devices and digitizing operators. Further, we ask whether combining these data introduces significant bias (i.e., change in means). We also extend these analyses to a procedure for the automated analysis of surfaces (Pomidor, Makedonska, & Slice, 2016), which does not require human digitization of landmarks. Finally, we investigate the effects of measurement error on the commonly used computation of phylogenetic signal. In doing this, we also evaluate the relative contribution of measurement error and phylogenetic uncertainty to variation in measured phylogenetic signal. To also gauge the effect of landmark choice, we perform landmark‐based analyses on two sets of landmarks: a “full” and a “reduced” set in which the most difficult to digitize landmarks have been removed. By showing how pervasive measurement error can be and which factors are its most important contributors, we hope to increase awareness on the implications of combining data from different sources.

## 2 MATERIALS AND METHODS

**Figure 1:**
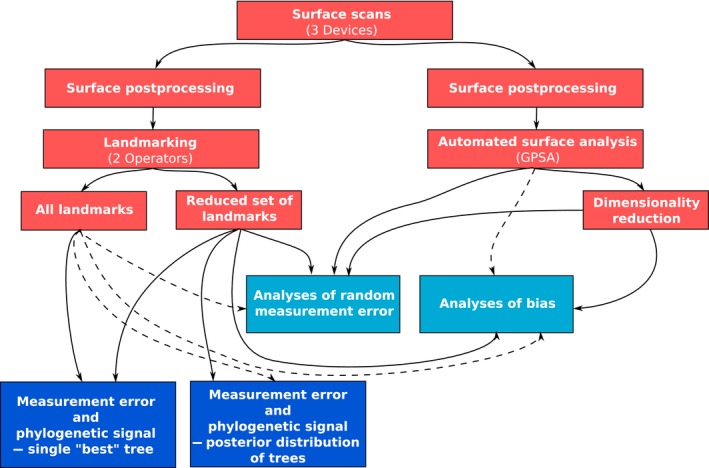
Schematic representation of the workflow of the present study. Red boxes represent data acquisition and preparation. Light blue boxes represent analyses of measurement error and bias. Dark blue boxes indicate analyses on the effect of measurement error on phylogenetic signal

A schematic representation of the workflow of the analyses in this study is presented in Figure 1.

### 2.1 Data acquisition and processing

We obtained 3D surface reconstructions from skulls (one skull per species) of 23 macropodoid marsupials, a group that includes kangaroos and wallabies (Table S1). These species were chosen based on preliminary evaluations of surface reconstructions to comprise a range of intermediate sizes large enough to obtain good scans across devices but small enough that differences in resolution could still be noticeable. For each skull, we obtained surface meshes using three different devices: two laser scanners and photogrammetry. The two laser scanners were a NextEngine 3D Ultra HD and a Solutionix Rexcan CS+, a commonly used laser scanner and a higher‐end device, respectively. Photogrammetry is a technique which allows surface models to be generated from photographs (Falkingham, 2012) and which is getting increasing attention from morphometricians (Aldridge, Boyadjiev, Capone, DeLeon, & Richtsmeier, 2005; Cardini, 2014; Muñoz‐Muñoz, Quinto‐Sánchez, & González‐José, 2016; Weinberg et al., 2009). We obtained photogrammetric models with a combination of a Nikon D5200 DSLR camera and the software Agisoft Photoscan (Agisoft LLC, St. Petersburg, Russia). Further details on devices, settings, and postprocessing can be found in the Appendix S1. In general, as these are very different devices and there are several choices that can influence the surface models obtained, we tried to make them comparable using the time spent to obtain each model (about one hour per scan) as a criterion.

Using the surface meshes thus obtained, two operators digitized independently with IDAV Landmark Editor (Wiley et al., 2005) a set of 31 type I landmarks (*sensu* Bookstein, 1991; Fig. S2), inspired by a previous study of macropod cranial variation (Milne & O'Higgins, 2002). These landmarks were chosen following a preliminary examination of surface scans where they were clearly visible (please, see the Appendix S1 for further details). The choice of using only type I landmarks (i.e., fixed landmarks on homologous points) was made to avoid the potentially confounding effect of using a sliding procedure (Bookstein, 1997; Gunz, Mitteroecker, & Bookstein, 2005) on semilandmarks.

For the subsequent analyses, each focal subset was subjected to generalized Procrustes analyses (Rohlf & Slice, 1990) in the R package *Morpho* (Schlager, 2016). For instance, when performing a comparison between Solutionix and NextEngine surface scans digitized by Operator 1, we combined the landmarks digitized by Operator 1 on Solutionix and NextEngine scans—and only those—and performed on this combined focal subset a single generalized Procrustes analysis. This analysis removes variation in translation, rotation, and scale in a set of landmark configurations. Using generalized Procrustes analysis on each focal subset guarantees the minimum possible shape distances among landmark configurations. On the contrary, using a single generalized Procrustes analysis on all the combinations of operators and devices combined prior to subsetting, distances between individual shapes might be larger.

To avoid a few particularly difficult landmarks affecting the conclusions of the study, the analyses were repeated excluding the seven (three bilateral landmarks, one on the midline) most problematic landmarks. These were chosen based on subjective reports from each operator where each operator ranked landmarks in order of perceived difficulty and then a consensus of the most difficult landmarks was drawn (see Appendix S1 for details). We will refer to this set of landmarks as “reduced.” Unless otherwise specified, all analyses were performed on the symmetric component of shape variation (Klingenberg, Barluenga, & Meyer, 2002; Klingenberg & McIntyre, 1998). Prior to specific analyses, preliminary principal component analyses (PCA) were performed and we produced scatterplots of the scores along the first two principal components, which were inspected for nonrandom patterns of dispersion. Similarly, scatterplots of scores along the first two between‐group principal components (species used as group) were used as an exploratory tool to visualize grouping of observations by species (as we used only one skull per species, all variation within species is due to operator and device). Between‐group PCA (Boulesteix, 2005) is an ordination technique increasingly used in geometric morphometrics (Firmat, Schliewen, Losseau, & Alibert, 2012; Franchini, Colangelo, Meyer, & Fruciano, 2016; Franchini et al., 2014; Fruciano, Franchini, Raffini, Fan, & Meyer, 2016; Fruciano, Pappalardo, Tigano, & Ferrito, 2014; Schmieder, Benítez, Borissov, & Fruciano, 2015), as the ordinations do not exaggerate the extent of separation between groups, which is one of the typical drawbacks of the commonly used scatterplots of canonical variate scores (Mitteroecker & Bookstein, 2011).

### 2.2 Levels of measurement error in landmark data

The relative amount of measurement error on the datasets (full and reduced configurations of landmarks, including all the operator/device combinations or only some of them) was measured using Procrustes ANOVA (Klingenberg & McIntyre, 1998; Klingenberg et al., 2002) in MorphoJ (Klingenberg, 2011). This approach partitions the total variation in aligned landmark coordinates (i.e., Procrustes residuals) into terms, allowing us to gauge the impact of variation among devices and operators relative to biological variation among individuals (species) and directional and fluctuating asymmetry. We also used the mean squares obtained from the Procrustes ANOVA (in this case only on the symmetric component of shape and using the “Individual” term as unique predictor) to compute an analogue of the intraclass correlation coefficient (also called “repeatability”; Arnqvist & Mårtensson, 1998), as described in Fruciano (2016).

### 2.3 Testing for bias in landmark data

Whether landmark data contain significant bias (i.e., nonrandom error) is a question distinct from how much variation is attributable to measurement error. Bias would be expected if systematic differences existed between devices or users. The question of whether significant bias is present can then be rephrased to ask whether a certain treatment (e.g., use of different device or operator) induces a change in mean. We investigated this question with a series of pairwise comparisons among surfaces digitized by the same operator (to test for bias due to device) and surfaces from the same device but digitized by the two operators (to test for bias due to operator digitization). We repeated this analysis using the dataset with all the landmarks and the dataset with a reduced number of landmarks. To test the null hypothesis of no difference in mean shape across repeated measures, we used a permutation test (1000 random permutations), permuting within subjects (see Appendix S1 for further information).

### 2.4 Use of automated methods of surface analysis

Recently, various methods that hold promise for decreasing the time necessary in acquiring data have been proposed. In particular, Pomidor et al. (2016) have proposed a new method to obtain from surface scans/models an analogue of Procrustes distance and perform superimpositions on a set of surfaces. This method has been implemented in the GPSA software (Pomidor et al., 2016), which outputs a set of principal coordinate scores obtained through principal coordinate analysis of the set of distances among surface models.

Here, we use this method on our set of scans from three different devices. To study how data acquired automatically from surfaces was affected by variation due to the device used, we computed the amount of measurement error (as repeatability) and tested for bias as described above for landmark data. We applied these analyses to the full set of principal coordinate scores obtained from the software GPSA and using a subset of principal coordinate scores, as determined using a dimensionality reduction approach. The dimensionality reduction was based on the observed explained variance of nonzero principal coordinates and the variance expected under a broken stick model (see Appendix S1 for details).

### 2.5 Measurement error and phylogenetic signal

As a statistic to quantify and test for phylogenetic signal we use Adams’ K_MULT_ (Adams, 2014), a recently proposed measure of phylogenetic signal which consists of a generalization of Blomberg's K statistic (Blomberg, Garland, & Ives, 2003) to multivariate data. As a reference phylogeny, we inferred a dated phylogeny based on a 33767‐base pair alignment of DNA sequences for 57 species (which we then pruned to match our morphometric data as appropriate) and a set of four node calibrations using a relaxed molecular clock (Drummond, Ho, Phillips, & Rambaut, 2006) in BEAST 1.8.3 (Drummond, Suchard, Xie, & Rambaut, 2012). In BEAST, we performed two independent runs of 20 million generations, sampled every 2000 generations, and discarded the first 20% as burn‐in. Employing this widely used software that integrates molecular dating over phylogenetic uncertainty with a few well‐supported calibrations reflects our effort to study the effect of measurement error in a typical phylogenetic comparative study, with realistic levels of phylogenetic uncertainty (see Appendix S1 for details).

**Figure 2:**
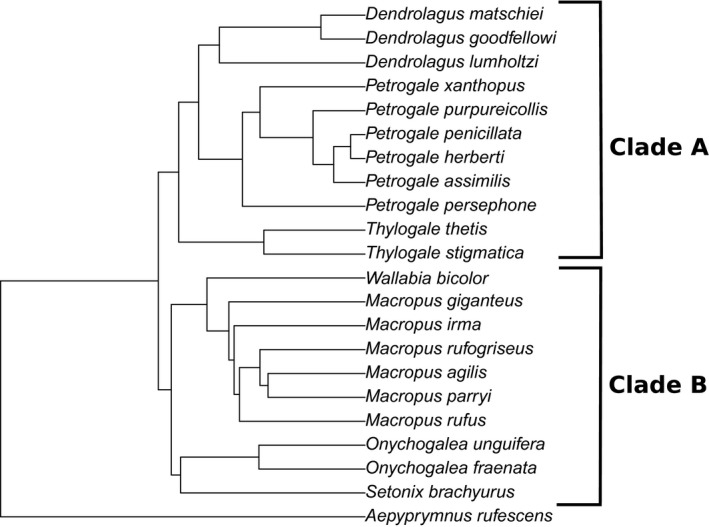
Phylogenetic tree used in analyses of phylogenetic signal, pruned to match the most comprehensive dataset used. Clade A and Clade B highlight two of the subsets used (see text and Appendix S1)

We investigated the interplay of measurement error and phylogenetic signal at two different levels. At the first level, we computed K_MULT_ for different subsets of our dataset using the best supported phylogeny from the posterior distribution (Figure 2, Fig. S3). This is the typical approach used in phylogenetic comparative studies. Specifically, we computed K_MULT_ for each unique combination of device and operator (three devices, two operators, for a total of six unique combinations) and then computed the coefficient of variation across the six K_MULT_ estimates. This analysis was performed on both the full dataset and the dataset excluding problematic landmarks. The analysis was repeated for the dataset comprising all the species in the phylogeny matching our morphometric dataset (Figure 2) and for four subclades. This allows us to verify the widespread assumption (Arnqvist & Mårtensson, 1998; Fruciano, 2016; Yezerinac et al., 1992) that, as the total variation in a sample is reduced (e.g., moving from interspecific to intraspecific samples or moving to shallower phylogenetic scales), measurement error will have stronger effect on inference (as the “signal‐to‐noise ratio” decreases). If this assumption were met in our sample, we would find a lower coefficient of variation in K_MULT_ in datasets comprising all the species compared to subsets. We extended this analysis by computing variation in K_MULT_ across device/operator combinations for random subsets of taxa in our phylogeny. This was done by randomly drawing a fixed number of taxa and computing on these taxa phylogenetic diversity (expressed as total branch lengths) with the package *caper* (Orme et al., 2013). For each of the six combinations of operator and device, these taxa were subjected to a Procrustes fit and the phylogenetic signal of each combination was computed as K_MULT_. Finally, the variation of K_MULT_ across different combinations of operator and device was expressed as coefficient of variation. The above algorithm was repeated 1000 times each for 5, 10, and 15 taxa and both landmarks sets (full and reduced).

In the second level of investigation, we incorporated phylogenetic uncertainty by computing K_MULT_ on each tree of the posterior distribution of trees (excluding the burn‐in). While estimating, reporting, and accounting for phylogenetic uncertainty is commonplace in phylogenetics and phylogenetic comparative studies (Felsenstein 1985, Huelsenbeck et al. 2000), investigations applying phylogenetic comparative approaches to geometric morphometric data typically use a single reference tree, thereby disregarding variation due to phylogenetic uncertainty and how this would affect inference. To ascertain the levels of variation in K_MULT_ due to phylogenetic uncertainty relative to variation in K_MULT_ due to measurement error (i.e., variation among devices and operators), we performed a resampling‐based version of analysis of variance (see Appendix S1 for details).

## 3 RESULTS

**Figure 3:**
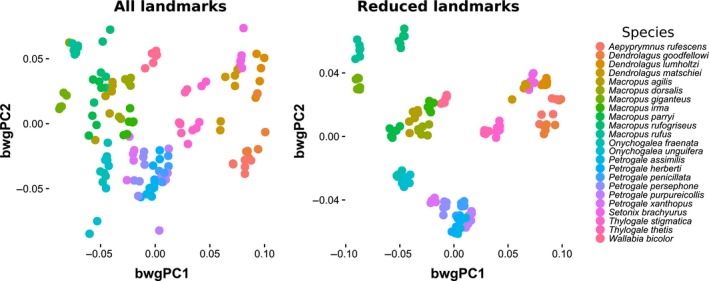
Scatterplot of the scores along the first two between‐group principal components (species used as group) for the dataset comprising all the landmarks and a dataset in which the most difficult landmarks had been removed

Scatterplots of the scores along the first two principal components on the full dataset (Fig. S4) show an apparent pattern of association between repeated measures of the same specimen and the second principal component. This pattern disappears in the dataset reduced to easily recognizable landmarks, where repeated measurements of the same specimens tend to cluster more tightly (Fig. S4). This pattern is confirmed by the scatterplots of the scores along the first two between‐group principal components (Figure 3). PCA scatterplots for residuals from species means show some nonrandom patterns associated with variation among devices and, even more clearly, variation among operators (digitization; Fig. S4).

### 3.1 Levels of measurement error in landmark data

In the Procrustes ANOVA of various datasets and their subsets (Tables 1, S2), the levels of measurement error are relatively low—but not trivial—when compared to the variation among species. The mean squares for the “Device” and “Operator” terms are, respectively, 1.7% and 2.1% of the mean squares for the “Individual” term in the dataset comprising all observations and all landmarks (Table 1). Device and operator explain, respectively, 5.4% and 10.2% of total variation (as computed by dividing the sum of squares for each term by the total sum of squares). This is also observed in subsets of the dataset including all the landmarks (Table S2). Variation between the two operators digitizing on the models obtained by a single device (Table S2) accounts between 8.09% (Solutionix scanner) and 12.06% (NextEngine scanner) of total variation and the mean squares for the term “Operator” is between 4.58% and 7.17% of the term “Individual” (variation among species). Variation between surface models digitized by the same operator for the dataset with all landmarks ranges between 9.22% and 11.25% of total variation (Table S2). This is confirmed by the value of repeatability for the dataset comprising all the landmarks, which is 0.83 in the full dataset (Table 1) and ranges between 0.78 and 0.88 in the various subsets (Table S2).

**Table 1:** Procrustes ANOVAs of various marsupial cranial datasets

Effect	SS	%Var	MS	*df*	*F*	*p*	Repeatability
Full dataset, all landmarks							
Individual (species)	0.965853	83.19789	0.000954	1012	65.87	<.0001	0.832
Side	0.000724	0.062351	1.81E‐05	40	1.25	.1415	
Individual × Side	0.012751	1.098381	1.45E‐05	880	0.91	.9638	
Device	0.063118	5.436964	1.6E‐05	3956	0.8	1	
Operator	0.118464	10.20441	2E‐05	5934			
Full dataset, reduced landmarks							
Individual (species)	0.910388	94.37447	0.001182	770	66.54	<.0001	0.961
Side	0.000742	0.076948	2.47E‐05	30	1.39	.0812	
Individual × Side	0.011728	1.215769	1.78E‐05	660	2.66	<.0001	
Device	0.01996	2.069179	6.68E‐06	2990	1.37	<.0001	
Operator	0.021836	2.263638	4.87E‐06	4485			

SS, sum of squares; %Var, percentage of variance accounted by the term (computed dividing the sum of squares for the term by the total sum of squares); MS, mean squares; *df*, degrees of freedom; *F*,* F*‐statistic; *p*,* p*‐value (parametric); repeatability, value of repeatability obtained using the formulas for the intraclass correlation coefficient on Procrustes ANOVA terms (see the text for details).

When compared to the terms related to directional and fluctuating asymmetry (i.e., “Side” and “Individual x Side”) in the analysis of the dataset comprising all landmarks, the terms “Device” and “Operator” have mean squares with similar order of magnitude and account for more variation (Table 1). This suggests that analyses of asymmetry could be unreliable.

Most importantly, simply eliminating landmarks that are difficult to digitize has substantial impact in reducing the level of measurement error. Indeed, in the full dataset with a reduced number of landmarks, the terms “Device” and “Operator” account for 2.07% and 2.26% of total variance and repeatability increases to 0.96 (Table 1). Similar proportions are obtained for subsets, where repeatability is 0.95 or higher (Table S2).

### 3.2 Testing for bias in landmark data

Our pairwise comparisons of repeated measurements showed a striking contrast between comparisons of datasets using all landmarks and comparisons of datasets using a reduced set of landmarks (Table 2). When using the dataset with all landmarks and comparing surfaces digitized by the same operator, only one test (i.e., between landmarks digitized by Operator 1 on NextEngine and photogrammetry surfaces) is significant. All the other comparisons, both of surfaces of different devices digitized by the same operator and of different operators digitizing surfaces from the same device, are not significant. On the other hand, all the comparisons using a reduced set of landmarks are significant, except the ones comparing photogrammetry and NextEngine surfaces (for both operators; Table 2).

**Table 2:** Significance of the test of bias for different subsets of our marsupial cranial data. The table reports *p*‐value based on a within‐subject permutation procedure (1000 random permutations). For comparisons between devices, *p*‐values above the diagonal were obtained with landmark sets digitized by Operator 1 and *p*‐values below the diagonal with datasets digitized by Operator 2. Significant comparisons in bold

	Between devices digitized by the same operator			Between operators, same device		
	Solutionix	NextEngine	Photogrammetry	Solutionix	NextEngine	Photogrammetry
All landmarks						
Solutionix	–	0.11	0.32	0.25	0.12	0.09
NextEngine	0.52	–	**0.04**			
Photogrammetry	0.19	0.17	–			
Reduced set of landmarks						
Solutionix	–	**<0.001**	**<0.001**	**<0.001**	**<0.001**	**<0.001**
NextEngine	**<0.001**	–	0.17			
Photogrammetry	**<0.001**	0.14	–			

### 3.3 Error and bias in automatically generated morphometric data

Plots of the first two principal coordinate scores as obtained by GPSA (Fig. S5) reveal a clustering of repetitions by species but also possible nonrandom patterns of variation associated with the device used to acquire the surface scans. The Procrustes ANOVA on the full set of principal coordinates reveals substantial variation due to device, accounting for about 28% of total variance, with a repeatability (as equivalent of the intraclass correlation coefficient) of 0.58 (Table 3). However, when using only the first five principal coordinates (chosen with a dimensionality reduction procedure), variation due to device accounts for less than 5 percent of total variance and repeatability increases to 0.95. When testing for bias, most of the pairwise comparisons of the same skulls acquired using different devices are significant (i.e., there is a variation in mean shape due to device; Table 3). However, the distances between skulls obtained using different devices are perceptibly lower when using only the first five principal coordinates (data not shown) and are not significant in the case of the comparison between surfaces acquired using the NextEngine scanner and photogrammetry (Table 3).

**Table 3:** Results of analyses of measurement error on data automatically acquired using GPSA with and without dimensionality reduction

	df	SS	MS	Rsq	*F*	*Z*	*p*	Repeatability
Procrustes ANOVA, full set of nonzero principal coordinates								
Species	23	11394.1	495.4	0.72365	5.1235	2.1345	.001	0.58
Residuals	45	4351.1	96.69					
Total	68	15745.3						
Procrustes ANOVA, first five principal coordinates								
Species	23	7061.6	307.024	0.96809	59.364	2.8411	.001	0.95
Residuals	45	232.7	5.172					
Total	68	7294.3						

	Solutionix	NextEngine	Photogrammetry					
*p*‐values for the pairwise tests of bias								
Solutionix	–	<0.001	<0.001					
NextEngine	0.02	–	0.006					
Photogrammetry	<0.001	0.172	–					

*df*, degrees of freedom; SS, sum of squares; MS, mean squares; Rsq, r squared; *p*,* p*‐value; in the pairwise test for bias, above the diagonal test based on the full set of nonzero principal coordinates and below the diagonal test based on the first five principal coordinates.

### 3.4 Measurement error and phylogenetic signal

**Figure 4:**
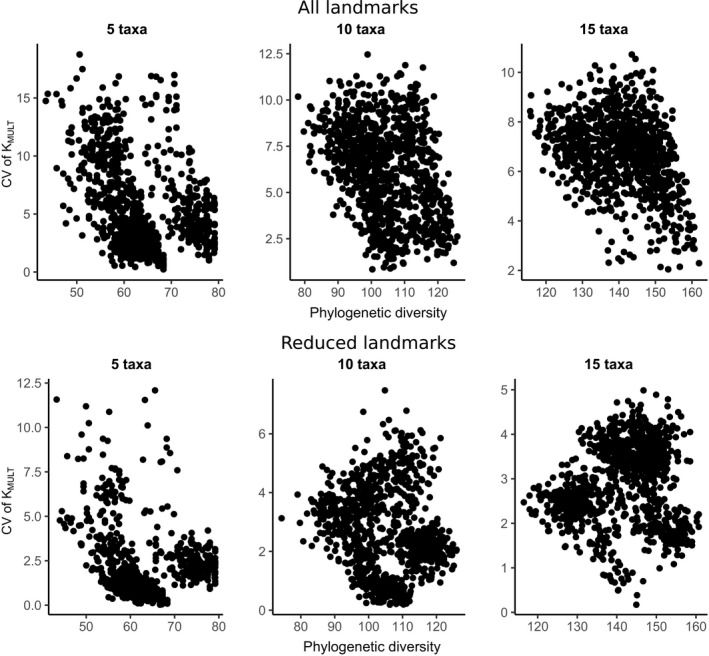
Plots of the coefficient of variation of K_MULT_ (across unique device/operator/landmark set combinations) against phylogenetic diversity for randomly drawn taxa (5, 10, 15)

We computed K_MULT_ based on a single reference tree for various datasets (Table S3) to test the expectation of higher variation in results at a shallower phylogenetic scale. Our results suggest that this expectation is not always met. Rather, the coefficient of variation for K_MULT_ across different operator/device combinations is almost always lower when going from a phylogenetically more diverse dataset to a dataset comprising only more similar species. When comparing for the same set of species the coefficient of variation between the full set of landmarks and the reduced set, the latter has lower variation (Table S3). In addition to this, K_MULT_ tends to be higher in the datasets with a reduced number of landmarks compared to their counterparts comprising all landmarks (Table S3). Extending the analysis to random subsets of taxa fails to reveal any clear association between the variation in K_MULT_ across operator/device combinations (expressed as coefficient of variation in K_MULT_) and phylogenetic diversity (Figure 4).

**Figure 5:**
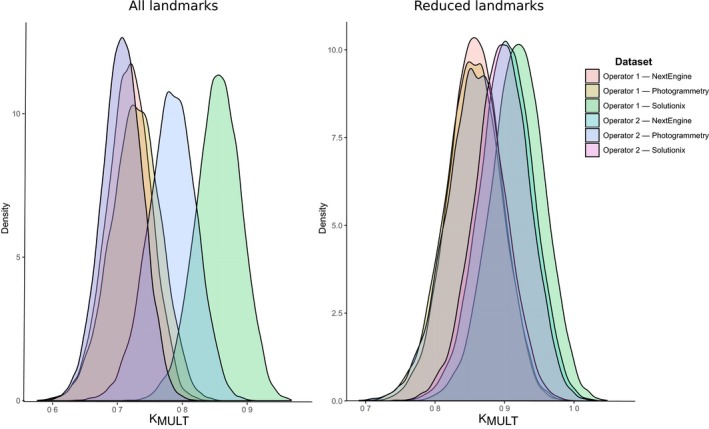
Distribution of the value of K_MULT_ for subsets (unique device/operator/landmark set combinations) computed using the posterior distribution of trees obtained from the phylogenetic analysis

Analyzing the values of K_MULT_ obtained using the full posterior distribution of trees to incorporate phylogenetic uncertainty further corroborates these results. In fact, for the most comprehensive set of landmarks, two distributions of K_MULT_ are clearly distinct from the other distributions but greatly overlap when excluding the most difficult landmarks (Figure 5). It is worth noticing that in some cases, the distribution of K_MULT_ changes not only in mean but also in shape. This is most apparent when focusing on the analyses on the various device/operator combinations for the genus *Macropus* when using all landmarks (Table S4). In these subsets, the standard deviation of K_MULT_ ranges between 0.004 and 0.017. More in general, 95% confidence intervals for K_MULT_ computed on the posterior distribution of trees for various subsets (Table S4) are as narrow as 0.015 and as wide as 0.212. Otherwise, computing K_MULT_ on the posterior distribution of trees for various subsets (Table S4) shows patterns broadly in agreement with the computations of K_MULT_ based on a single “best” tree (Table S3). Indeed, both the mean and the median of K_MULT_ are generally higher when excluding the most problematic landmarks.

We performed ANOVAs on the value of K_MULT_ for random subsamples of the distributions to gauge the relative contribution of phylogenetic uncertainty and measurement error to variation in K_MULT_ estimates. Our results (Table S5) quantitatively confirm the observations on distributions of K_MULT_. In fact, excluding the most difficult landmarks generally results in a sharp increase of the proportion of variance accounted for by the term “Tree” (which we interpret as variation in K_MULT_ due to phylogenetic uncertainty) relative to the proportion of variance accounted for by the other terms (which reflect variation in K_MULT_ due to measurement error). However, while in some cases the “Tree” term explains the clear majority of variance in K_MULT_, in most cases variation in K_MULT_ due to other terms (i.e., due to measurement error) is nontrivial (Table S5).

## 4 DISCUSSION

Here we have analyzed measurement error in 3D geometric morphometrics, with a focus on the situation of combining data obtained from different devices or operators. We have explored three main areas: (1) the existence and the extent of both random measurement error and bias in landmark‐based geometric morphometrics, (2) the extent of measurement error and bias in automatically generated geometric morphometric data, and (3) the sensitivity of a commonly used measure of phylogenetic signal to realistic levels of measurement error. A descriptive summary of the results can be found in Table 4.

**Table 4:** Descriptive summary of the results

Analysis	Results
Levels of error (human‐digitized landmarks)	Using all landmarks, measurement error accounts for about 10% of total variance (repeatability around 0.8) Removing landmarks difficult to digitize, measurement error accounts for 1%–4% of total variance (repeatability usually >0.95) Effect size of measurement error of the same order of magnitude as asymmetric components Error due to digitizing operator higher than error due to device
Presence of bias (human‐digitized landmarks)	Using all landmarks, generally no significant bias Removing landmarks difficult to digitize, bias is generally significant
Levels of error (automated method)	Using all the nonzero principal coordinates, error accounts for almost 30% of variance (repeatability 0.58) Performing dimension reduction, error accounts for less than 5% of variance (repeatability 0.95)
Presence of bias (automated method)	Significant bias generally present
Measurement error and phylogenetic signal, single tree	In some cases, the value of K_MULT_ for unique operator/device combinations is more variable at a broader than at a shallower phylogenetic scale (K_MULT_ differences between subsets between 0.01 and 0.18). No clear association of phylogenetic diversity and variation in K_MULT_ estimates across operator/device combinations for random samples of taxa. When reducing measurement error by eliminating the landmarks which are hardest to digitize, phylogenetic signal increases
Measurement error and phylogenetic signal, posterior distribution of trees	When using all landmarks, typically 60%–80% of variance due to error When using the reduced set of landmarks, 70%–95% of variance due to phylogenetic uncertainty

### 4.1 Levels of measurement error in landmark data

Our results highlight the importance of landmark choice. Excluding from the analyses a few landmarks that the operators found harder to digitize generally resulted in an impressive reduction of measurement error. This result is, in part, expected, but it points out an important issue. The difficulty in digitizing landmarks could depend on the individual operator and on the samples, so relying heavily on published or existing landmark sets can produce unwanted levels of measurement error if the new operator finds the landmarks difficult to digitize.

It is also interesting to notice that in our analyses, a much larger amount of variance was explained by the operator compared to the device. If this pattern were common, this would mean that—when provided with the choice—it is better to combine existing surface scans and have a single operator to digitize landmarks than combining existing sets of landmarks, even if obtained from the same device. However, a recent study on a small intraspecific sample of wolf skulls comparing surface scans and photogrammetric surfaces (Evin et al., 2016) has reported the opposite pattern (higher proportion of variance due to device than due to digitization). Clearly, in the more common case of combining landmark sets digitized by multiple operators on surface scans obtained from multiple devices, both sources of variation will be present in the final dataset.

The error components of variance are also in the same order of magnitude—and often larger—than the components reflecting asymmetry (Side and Individual x Side). This means that combining different datasets or surfaces for studies of asymmetry can be particularly problematic. The idea of asymmetry being potentially heavily affected by measurement error is certainly not new (Fruciano, 2016; Klingenberg et al., 2010; Leamy & Klingenberg, 2005). However, here we show empirically that this is the case for the error due to variation among operators and devices. We imagine that this pattern may be quite general, except perhaps in cases of a very large asymmetric component.

### 4.2 Bias in landmark data

We show that bias can be pervasive and that significant bias is often detected when appropriate statistical procedures are used for testing. This reinforces the suggestion (Fruciano, 2016) that the presence of bias in geometric morphometric datasets has previously gone unnoticed either because of lack of testing or due to inappropriate statistical procedures (i.e., using permutation schemes designed for independent observations, as opposed to permuting within subjects as we did). Furthermore, in most cases, bias only becomes significant when removing the landmarks that are more difficult to digitize. In other words, when a large amount of probably random variation due to certain landmarks is removed, subtler differences due to nonrandom variation between operators and devices become apparent. This bias is unlikely to cause serious problems because it accounts for a small proportion of variance. However, this nonrandom variation could be incorporated in inference if care is not taken. For instance, if one combined data for two populations of the same species, with each population digitized by a different operator and then tested for difference in mean shape between the two populations, then differences due to operator—minor as they might be—would be “mixed” with true biological differences between populations.

### 4.3 Error and bias in automatically generated morphometric data

Our analyses of automatically generated morphometric data obtained with GPSA (Pomidor et al., 2016) provided some surprising results. A reasonable assumption is that automated methods perform worse than data digitized by human operators. This assumption is clearly met when using all the nonzero dimensions produced by GPSA using a principal coordinate analysis of distances, which have poor repeatability. However, this does not apply when a dimensionality reduction is used, with levels of error similar to the ones observed in the more error‐free human‐digitized datasets. Interestingly, when using a similar dimensionality reduction approach on the landmark datasets, we did not observe an improvement in repeatability (first five principal components of the full configuration: repeatability 0.79; first four components of the reduced configuration: repeatability 0.95; see Table 1 for the repeatabilities obtained without dimensionality reduction). In addition to this, the dimensionality reduction procedure results in a reduction of bias and lack of its significance in one case. This suggests that the method implemented in GPSA might be a promising alternative to human landmarking of surface scans when surfaces from different sources are combined, if used in combination with dimensionality reduction as suggested by its authors. The high repeatability of the GPSA method when followed by dimension reduction most likely comes at the cost of substantial loss of information on fine details of surfaces. However, this might be acceptable in situations where larger‐scale shape variation is of interest. It is also important to note that the consequences and effectiveness of dimension reduction might depend on the sample and on the method of dimension reduction used. In current geometric morphometrics, analyzing the full dimensional (tangent) shape space is preferred and dimensionality reduction should be approached with caution. It is also unclear whether dimensionality reduction has reduced the measurement error due to the use of different devices or variation introduced by the GPSA procedure itself. Further, it is worth noting that these findings on GPSA do not necessarily generalize to other methods for the automated acquisition of morphometric data. In fact, previous studies on other automated methods (Gonzalez, Barbeito‐Andrés, D'Addona, Bernal, & Perez, 2016) have shown these can compare poorly to human‐assisted digitization of landmarks.

### 4.4 Measurement error and phylogenetic signal

As a further aim, we set out to understand how variation due to measurement error affects the results of downstream statistical analyses, and in particular the estimation of phylogenetic signal. To this aim, we measured phylogenetic signal as K_MULT_ (Adams, 2014), a recently proposed—and increasingly popular—metric which generalizes Blomberg's K (Blomberg et al., 2003) to multivariate data. This statistic and its use in hypothesis testing has a number of attractive properties, including insensitivity to dimensionality, appropriate type I error rate, and high power (Adams, 2014). Here, the question is whether and to what extent the estimation of phylogenetic signal is affected by measurement error and how the variation produced by measurement error compares to other sources of variation and uncertainty. Phylogenetic uncertainty is an obvious source of uncertainty in phylogenetic comparative analyses, but, at the same time, it is often neglected in geometric morphometric studies. Further, we could also test empirically the widely held assumption of a stronger effect of measurement error on statistical inference at shallower phylogenetic scales.

Our results only partially conform to this expectation. In fact, variation among estimates of phylogenetic signal in different datasets for a single reference phylogeny was in some cases lower in subclades (e.g., in *Macropus*) than in the full dataset. This could be explained by measurement error, especially at certain landmarks, accumulating more in certain clades than in others and, generally, interacting with variation in biological features unpredictably. The same analysis showed that in most cases, the reduced set of landmarks had higher phylogenetic signal. We hypothesize that reduced measurement error due to the removal of problematic landmarks “exposes” more of the true, underlying, phylogenetic signal. Downward biased estimates of phylogenetic signal due to unaccounted intraspecific variation (whether due to biological variation or measurement error) have also been supported by simulations of univariate traits (Ives, Midford, & Garland, 2007). The absence of a clear relationship between phylogenetic diversity and variation in estimates of K_MULT_ was also found when using the same rationale on random subsets of taxa in the phylogeny.

When computing K_MULT_ on distributions of trees so as to compare variation due to phylogenetic uncertainty and measurement error, a range of different situations occurred, probably reflecting local levels of phylogenetic uncertainty and error. This further reinforces suggestions that measurement error, phylogenetic uncertainty, and biologically relevant variation can interact unpredictably. The most frequent pattern, however, was a relatively large effect of measurement error in the datasets with all landmarks. By contrast, measurement error was reduced with fewer landmarks and variation due to phylogenetic uncertainty became dominant. Thus, measurement error can have a substantial impact on estimates of K_MULT_ but moderate levels of phylogenetic uncertainty in both topology and branch lengths most often have a reduced impact on K_MULT_. Then, while K_MULT_ generalizes well to different numbers of dimensions and the main conclusions drawn from using K_MULT_ in hypothesis testing are quite stable (they were generally significant, data not shown), the comparison of values of K_MULT_ across different studies or datasets could be affected by measurement error.

### 4.5 How to address measurement error? Strategies and conclusions

Two nonmutually exclusive approaches are available to address measurement error when combining data from multiple sources: accounting for and reporting error. Discussing this at length is beyond the scope of this study (see previous extended discussions in Arnqvist and Mårtensson 1998 and Fruciano 2016). However, random measurement error is often reduced by averaging repeated measurements (Arnqvist & Mårtensson, 1998; Fruciano, 2016). When measurement error has precise directions in shape space which can be modeled (even based on a subset of specimens during a preliminary study), it can often be removed from the data. This strategy—which is accomplished by projecting observations to the subspace orthogonal to a given vector in multivariate space (Gharaibeh, 2005; Valentin, Penin, Chanut, Sévigny, & Rohlf, 2008)—has been fruitfully used on empirical datasets to remove artefactual variation due to position of the head in pictures of human faces (Gharaibeh, 2005) and body arching in fish (Franchini et al., 2014; Fruciano, Tigano, & Ferrito, 2011, 2012; Fruciano, Franchini, Kovacova, et al., 2016; Ingram, 2015; Valentin et al., 2008), as well as variation due to sexual dimorphism (Fruciano et al., 2014). Similar procedures could also be used to estimate the amount of variation realistically attributable to measurement error. This could be especially useful in cases when measurement error is collinear with biologically relevant variation (i.e., has the same direction in shape space) and cannot be removed from a dataset. In this case, it might be possible to at least derive confidence intervals for estimates of parameters obtained in downstream statistical analyses. Here, we have used estimation of K_MULT_ on a sample of trees from the Bayesian posterior distribution of trees obtained in phylogenetic inference to obtain estimates of variation of this statistic due to phylogenetic uncertainty. We also provide the R code for this in the Supplementary Material, to facilitate computations of the variation due to phylogenetic uncertainty similar to ours. This is a relatively crude method to estimate variation due to phylogenetic uncertainty and it is likely that more refined approaches will be developed in the future.

To conclude, as we have highlighted that measurement error can be a source of substantial variation when combining different morphometric datasets and can have a sometimes unexpected effect on parameter estimates, we want to point out that we do not have an “all or nothing” perspective on measurement error. Estimating measurement error might not always be possible. The time spent to estimate measurement error could also be spent in generating more data, thereby potentially increasing statistical power, or making certain large‐scale analyses simply possible. These are all considerations that have to be made in a case‐by‐case cost–benefit analysis. However, researchers willing to combine different datasets should at least consider the issue of measurement error and its potential impact on their inference. In most practical situations, the common suggestion of a preliminary study of measurement error on a small subset of specimens (Arnqvist & Mårtensson, 1998; Fruciano, 2016) represents a good compromise between costs and benefits.

## DATA ACCESSIBILITY

Data available from the Dryad Digital Repository: http://dx.doi.org/10.5061/dryad.t9888

## CONFLICT OF INTEREST

None declared.

## AUTHOR CONTRIBUTIONS

CF designed the study, acquired part of the data, performed all the analyses, and wrote a first draft of the manuscript. MAC, KB, and TD acquired most of the data and contributed to the manuscript. VW contributed to study design and to the manuscript. MJP contributed data and fossil calibrations to the phylogenetic analysis and contributed to the manuscript.
