# Supplementary figures and images for: Sharing is caring? Measurement error and the issues arising from combining 3D morphometric datasets
Source: Ecol Evol. 2017 Jul 31;7(17):7034–46. doi: 10.1002/ece3.3256 (PMC5587461; doi:10.1002/ece3.3256)

Number of datasets

30 -

20 -

10 -

0 -

2000

2005

2010

2015

Year

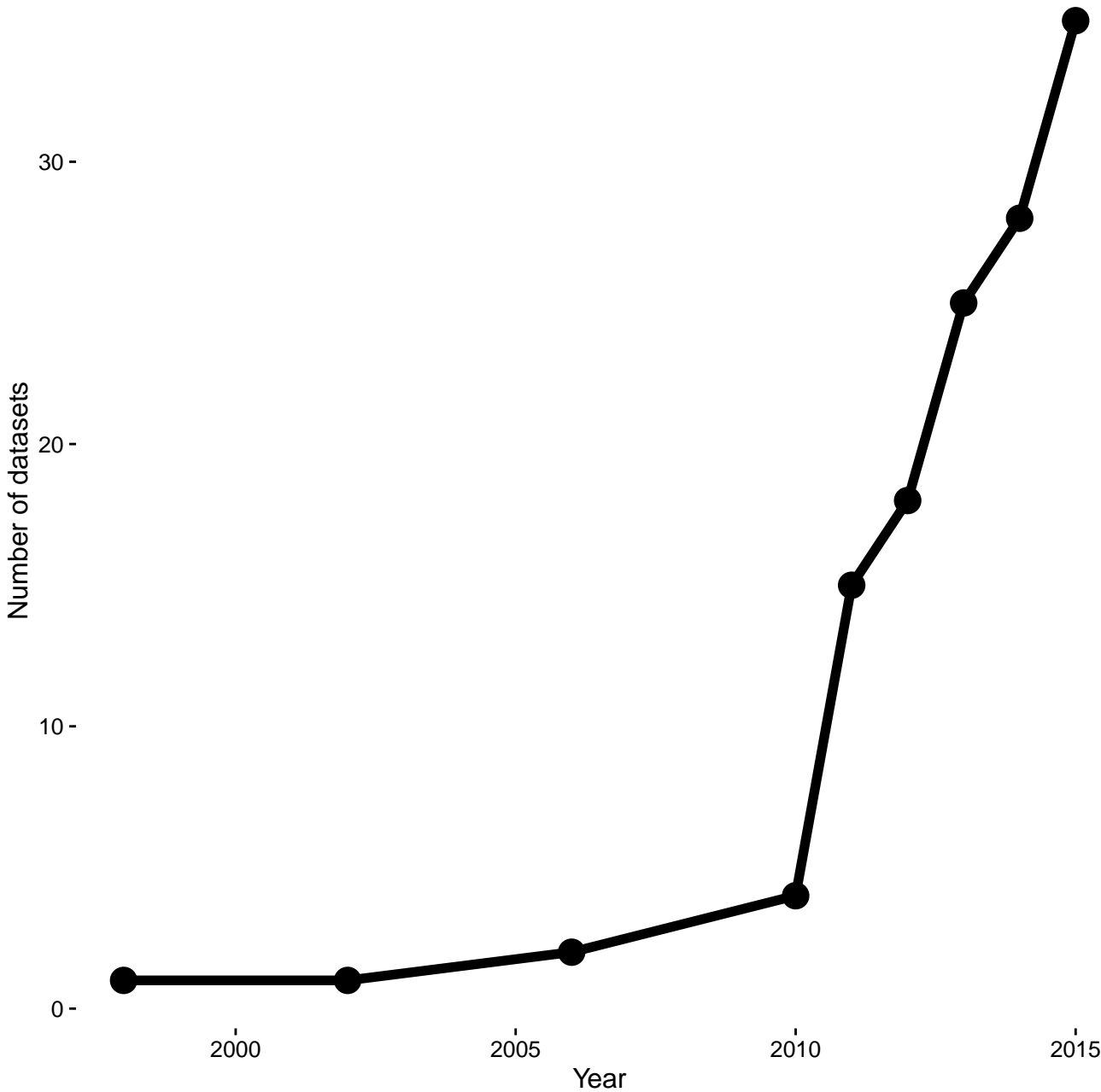

Supplement: Supplementary file 1 [file ECE3-7-7034-s001.pdf]

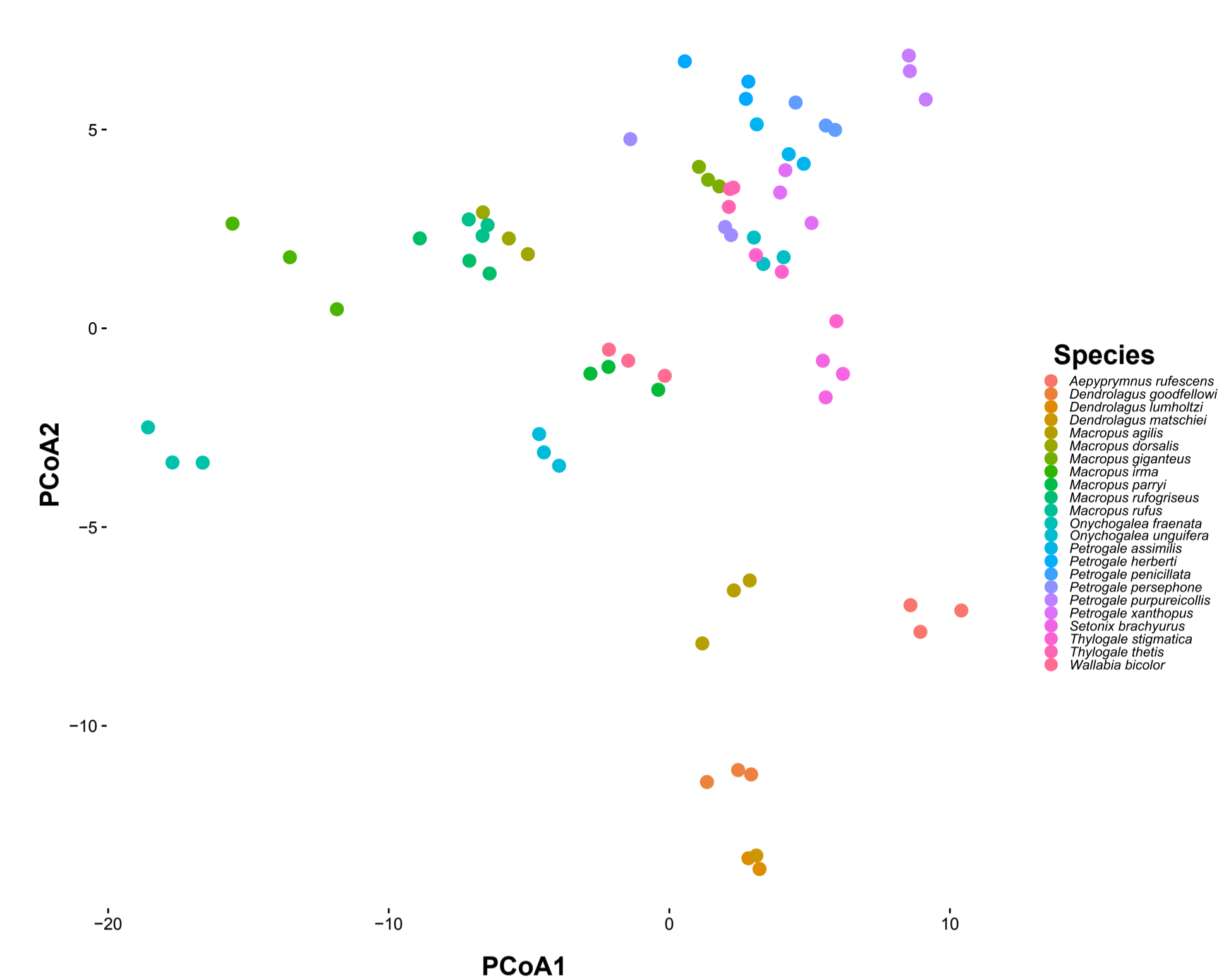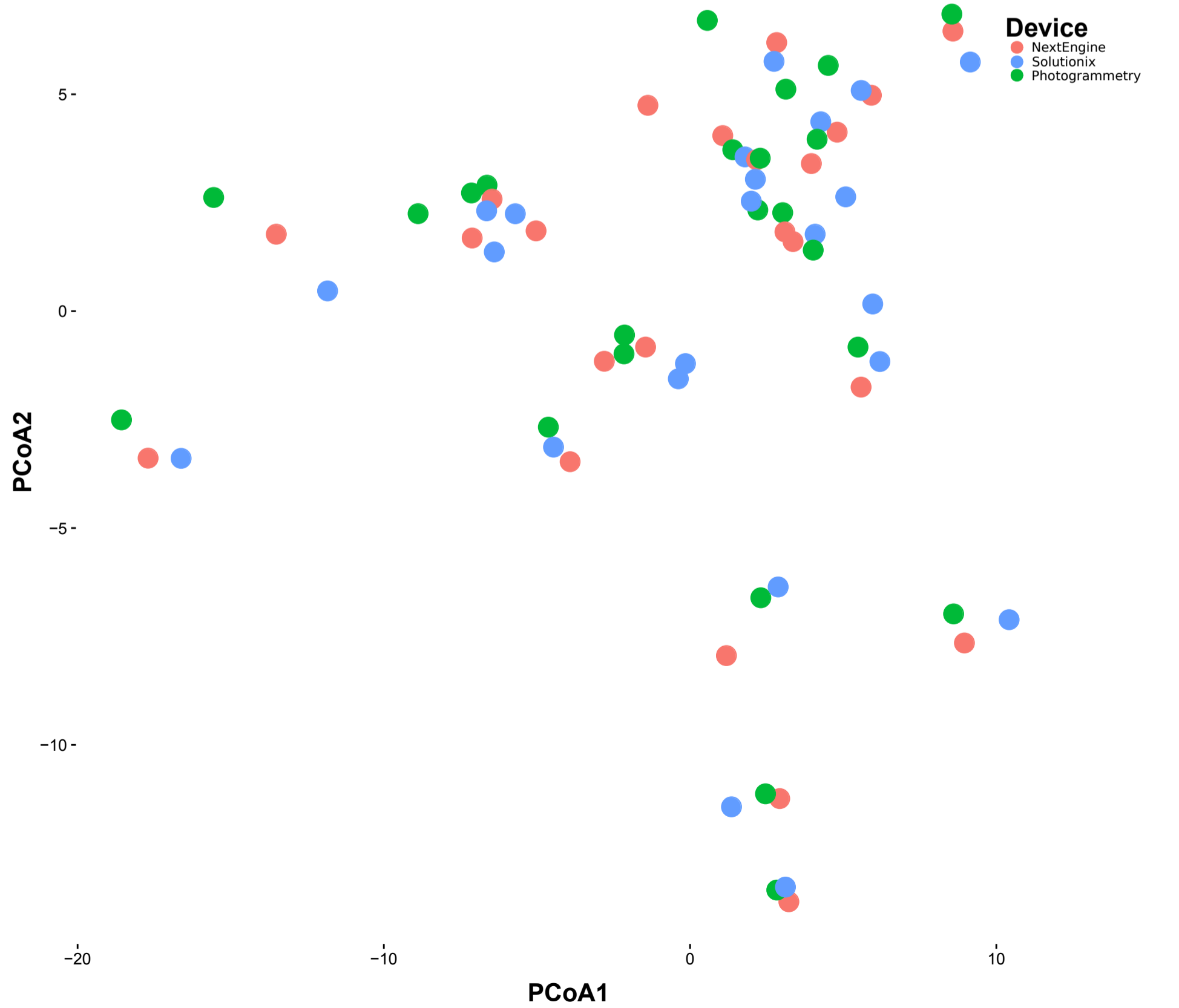

Supplement: Supplementary file 5 [file ECE3-7-7034-s005.pdf]
