# Supplementary material for: Sharing is caring? Measurement error and the issues arising from combining 3D morphometric datasets
Source: Ecol Evol. 2017 Jul 31;7(17):7034–46. doi: 10.1002/ece3.3256 (PMC5587461; doi:10.1002/ece3.3256)
Supplement: Supplementary file 2 [file ECE3-7-7034-s002.pdf]

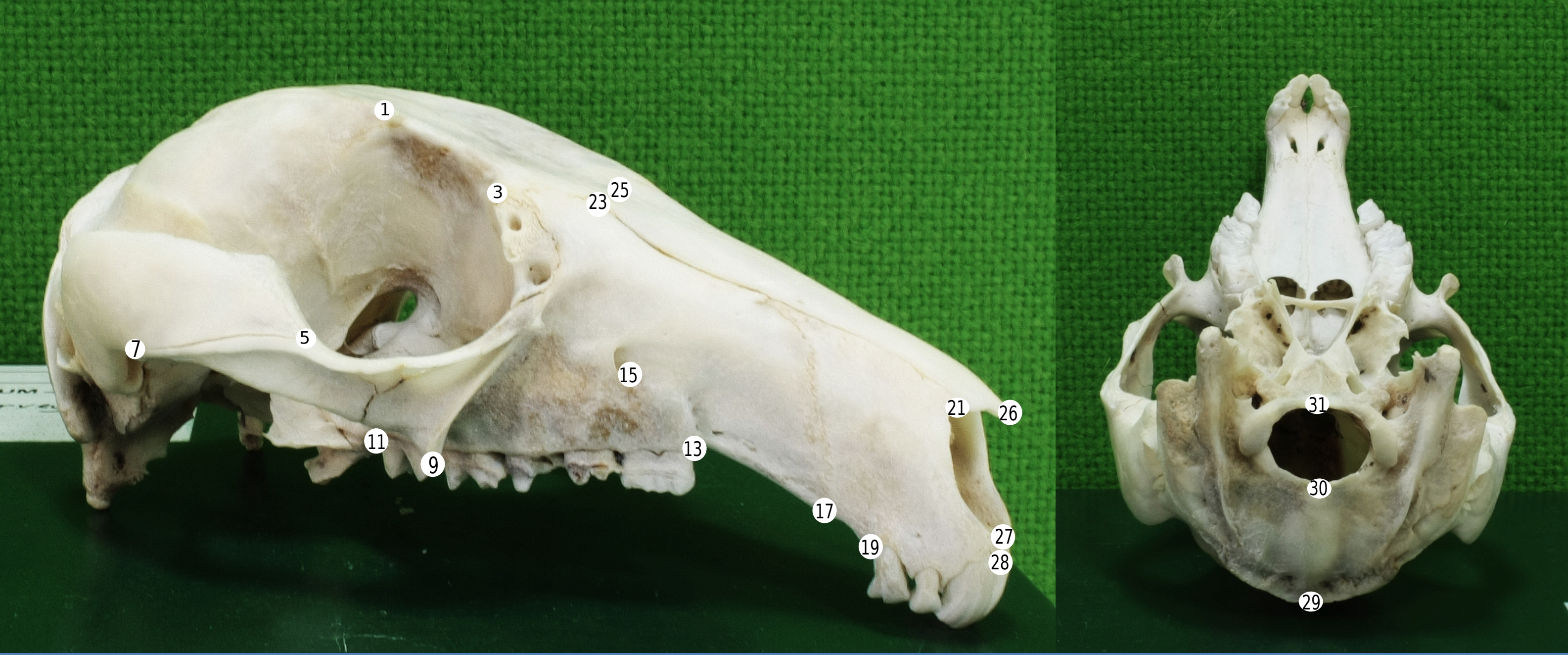

Landmark(s)	Definition
1&2	Fronto-parietal suture at the intersection of the temporal crest
3&4	Fronto-lachrymal suture at the medial orbital margin
5&6	Anterior point of the zygomatic process of the temporal bone
7&8	Posterior end of the zygomatico-temporal suture
9&10	Lowest point on the anterior end of the zygomatic arch `Masseteric process'
11&12	Alveolar margin at the posterior aspect of the last molar
13&14	Alveolar margin at the anterior aspect of the first cheek tooth
15&16	Infraorbital *foramen* (ventral margin of the *foramen*; in the species with two openings, the largest)
17&18	Maxillary-premaxillary suture at the alveolar margin
19&20	Aveolar margin at the posterior aspect of the 3rd incisor
21&22	Naso-premaxillary suture at the margin of the nasal aperture
23&24	Fronto-naso-maxillary junction
25	Naso-frontal suture in the midline
26	Tip of the nasal bones in the midline
27	Inter-premaxillary suture at the inferior margin of the nasal aperture
28	Inter-premaxillary suture at the alveolar margin
29	Occipital tip (i.e., along the midline, the most posterior and highest point)
30	Posterior (dorsal) extremity of *foramen magnum*
31	Anterior (ventral) extremity of *foramen mangum*
