# Supplementary material for: Sharing is caring? Measurement error and the issues arising from combining 3D morphometric datasets
Source: Ecol Evol. 2017 Jul 31;7(17):7034–46. doi: 10.1002/ece3.3256 (PMC5587461; doi:10.1002/ece3.3256)
Supplement: Supplementary file 4 [file ECE3-7-7034-s004.pdf]

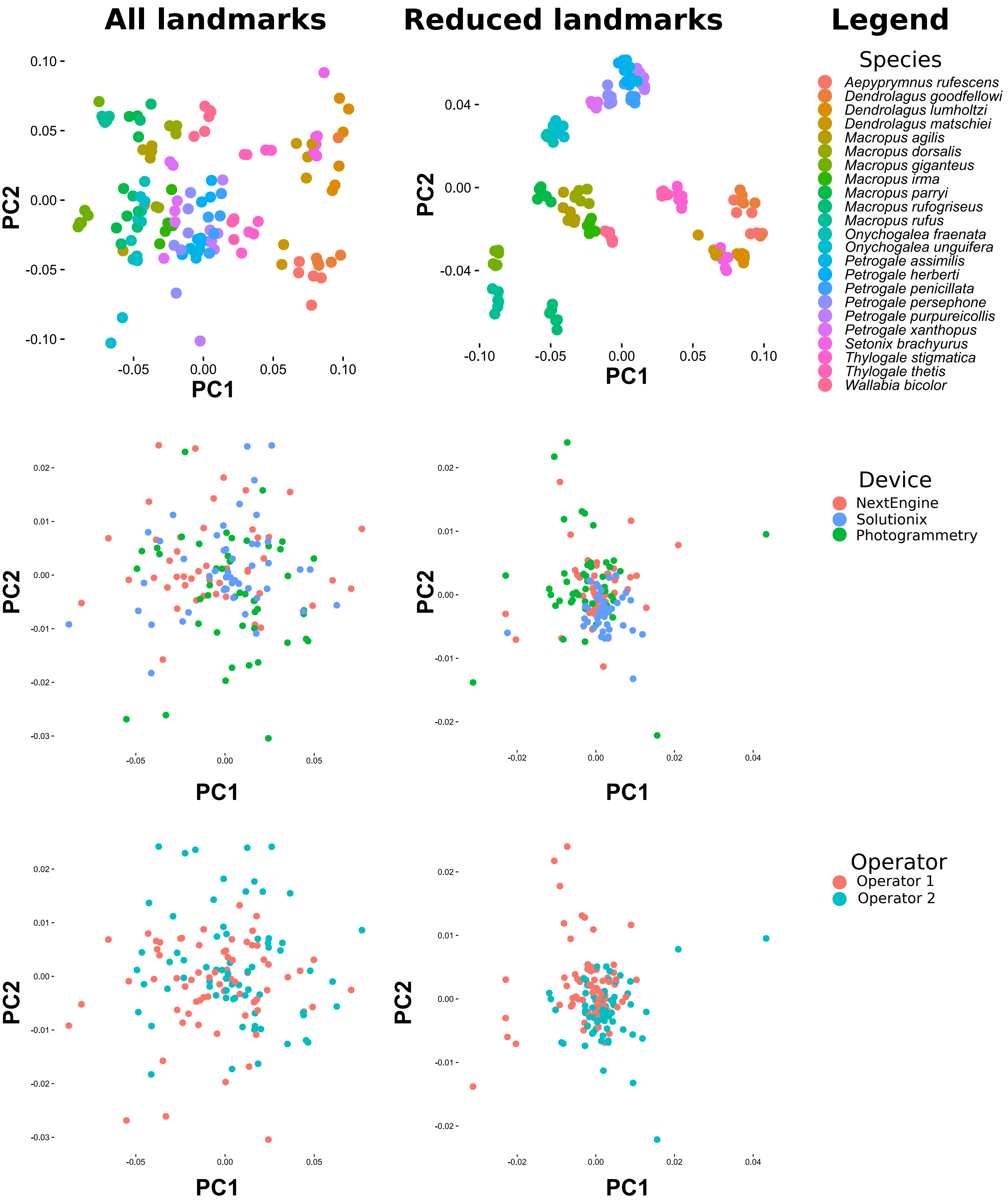

All landmarks
Reduced landmarks
Legend
PC1
PC2
Species
Aepyprymnus rufescens
Dendrolagus goodfellowi
Dendrolagus lumholtzi
Dendrolagus matschiei
Macropus agilis
Macropus dorsalis
Macropus giganteus
Macropus irma
Macropus parryi
Macropus rufogriseus
Macropus rufus
Onychogalea fraenata
Onychogalea unguifera
Petrogale assimilis
Petrogale herberti
Petrogale penicillata
Petrogale persephone
Petrogale purpureicollis
Petrogale xanthopus
Setonix brachyurus
Thylogale stigmatica
Thylogale thetis
Wallabia bicolor
Device
NextEngine
Solutionix
Photogrammetry
Operator
Operator 1
Operator 2
