# Supplementary material for: Sharing is caring? Measurement error and the issues arising from combining 3D morphometric datasets
Source: Ecol Evol. 2017 Jul 31;7(17):7034–46. doi: 10.1002/ece3.3256 (PMC5587461; doi:10.1002/ece3.3256)
Supplement: Supplementary file 6 [file ECE3-7-7034-s006.docx]

| **Supplementary Table I**. Specimens used in the present study, all obtained from the Queensland Museum. | |
| --- | --- |
| **Species** | **Voucher code** |
| \| *Aepyprymnus rufescens* \| \| --- \| \| *Dendrolagus goodfellowi* \| \| *Dendrolagus matschiei* \| \| *Macropus agilis* \| \| *Macropus dorsalis* \| \| *Macropus giganteus* \| \| *Onychogalea fraenata* \| \| *Onychogalea unguifera* \| \| *Petrogale assimilis* \| \| *Petrogale penicillata* \| \| *Petrogale persephone* \| \| *Petrogale purpureicollis* \| \| *Petrogale xanthopus celeris* \| \| *Thylogale thetis* \| \| *Wallabia bicolor* \| \| *Dendrolagus lumholtzi* \| \| *Macropus irma* \| \| *Macropus parryi* \| \| *Macropus rufogriseus* \| \| *Petrogale herberti* \| \| *Setonix brachyurus* \| \| *Macropus rufus* \| \| *Thylogale stigmatica* \| | \| JM20274 \| \| --- \| \| JM18691 \| \| JM6432 \| \| J10763 \| \| J3826 \| \| 19568 \| \| J6277 \| \| JM20395 \| \| JM10474 \| \| JM17637 \| \| JM11192 \| \| JM20293 \| \| J4468 \| \| JM16351 \| \| J21546 \| \| JM19318 \| \| J10997 \| \| JM5762 \| \| JM15073 \| \| J4892 \| \| J17423 \| \| 16682 \| \| J3251 \| |
