# Supplementary material for: Sharing is caring? Measurement error and the issues arising from combining 3D morphometric datasets
Source: Ecol Evol. 2017 Jul 31;7(17):7034–46. doi: 10.1002/ece3.3256 (PMC5587461; doi:10.1002/ece3.3256)
Supplement: Supplementary file 14 [file ECE3-7-7034-s014.docx]

Supplementary methods

**Acquisition of surfaces and post-processing**

We obtained 3D surface reconstructions from a set of 23 Macropodid Marsupial skulls (Supplementary Table I) using three different devices: a Solutionix Rexscan CS+ laser scanner, a NextEngine 3D Ultra HD laser scanner and photogrammetry (using Agisoft Photoscan on digital photographs taken with a Nikon D5200 DSLR camera). The NextEngine laser scanner is possibly the most commonly used laser scanner in morphometrics, its popularity stemming from its relatively low cost and portability. The Solutionix scanner is a higher-end scanner, which is more expensive than the NextEngine scanner, which guarantees a higher resolution but is not portable. Although these devices are quite different, we tried to match each acquisition pipeline so that the effort spent on each device was similar. The Solutionix scanner employed the EzScan7 software and had its medium field of view lens (200 mm). Scans with the Solutionix scanner were obtained at 4 skull positions (dorsal, ventral, and two laterals views) using for each position 40 scans. The NextEngine scanner was used via the NextEngine ScanStudio software. NextEngine scans were completed using the following settings: macro, 360 degree scan, 8 divisions/panels per scan, a target setting of 'neutral' and 1200 (lowest of the HD settings) points per inch. The 1200 points per inch setting was used because the higher HD setting created too much noise on the mesh. A minimum of two scans, using two different skull positions, were used per specimen to ensure all parts of the specimens were scanned. For larger specimens, such as *Macropus,* scans of additional panels were used as needed. Scans for each specimen were then aligned and fused in NextEngine ScanStudio. Photogrammetric reconstructions were obtained using a Nikon D5200 DSLR camera with a tripod and a turntable where the object was placed. The camera was fitted with a Nikon 85mm 1:3.5G micro lens. 90-110 pictures per specimen were used for photogrammetric reconstruction in Agisoft Photoscan.

We kept post-processing of the surface models to a minimum to avoid post-processing affecting the results. In particular, we performed a conversion of the STL files obtained into PLY files using the software meshconv by Patrick Min (<http://www.patrickmin.com/meshconv/> ), which includes a step to automatically remove duplicate vertices. In the case of the Solutionix surface models, we also decimated the surfaces to 3 million faces (which is consistent with the number of faces produced by the NextEngine scanner) using a batch script in Meshlab (Cignoni et al. 2008). This was necessary to make the surface scans more tractable and amenable to landmark digitization in normal desktop computers.

**Acquisition of landmark data**

Using the surface meshes, two operators digitized independently a set of 31 type I landmarks (*sensu* (Bookstein 1991); Supplementary Fig. 1), inspired by a previous study of macropod cranial variation (Milne and O'Higgins 2002).

While the two operators were not strictly independent, care was taken to simulate a situation akin to sharing of morphometric data. In particular, the set of landmarks was agreed upon *a priori* based on the examination of three skulls and their preliminary scans and on a set of landmarks previously used in a biological study. Operator 1 did not have access to the skulls after this stage, whereas Operator 2 acquired surface scans using the Solutionix laser scanner. After agreeing on a set of landmarks and on details of their definitions, the operators, digitized the set of landmarks independently (thus simulating a situation in which data is shared in the form of landmark coordinates) and without accessing the skulls (thus simulating a situation in which data is shared in the form of surface scans). As both operators reported problems with certain landmarks and certain scans, they were asked to provide – independently and blindly to the answer of the other operator – a list of the most problematic landmarks. Then, based on these reports, all the analyses performed in this study were repeated excluding the seven (three bilateral – 1, 2, 3, 4, 17, 18 – one along the midline, 28) most problematic landmarks. This step was taken to avoid that our results would depend on a few very problematic landmarks or specimens but at the same time maintaining any inherent, general, limitation. Sets of landmark coordinates were aligned with a generalized Procrustes analysis in the R package *Morpho* (Schlager 2016).

**Preliminary PCAs and between-group PCAs**

Prior to specific analyses, a set of principal component analyses (PCA) was performed on the symmetric component of shape and on residuals from species means (i.e., variation due to repetition) for the symmetric component. These, were performed both on the full dataset and on the dataset with a reduced number of landmarks, for a total of four different PCAs. Scatterplots of the scores along the first two components were then produced using the R package *ggplot2* (Wickham 2009) to inspect them for non-random patterns. Between-group PCAs were performed on the symmetric component of the full dataset and on the symmetric component of the dataset with a reduced number of landmarks.

**Levels of measurement error**

Levels of random measurement error were measured using Procrustes ANOVA. Specifically, to quantify the relative amounts of variation among individuals, side, individual x side interaction and repetition, we performed a Procrustes ANOVA (Klingenberg and McIntyre 1998, Klingenberg et al. 2002) in MorphoJ (Klingenberg 2011). We also computed values of the analogue of the intraclass correlation coefficient (repeatability) described in Fruciano (2016). Briefly, this approach consists into applying the formulas for univariate repeatability to the mean squares obtained in Procrustes ANOVA (Fruciano 2016). This was obtained by performing a Procrustes ANOVA on the symmetric component of shape variation after projection to the tangent space using the package *geomorph* to compute the mean squares estimates. This approach was chosen as often investigators are interested in the symmetric component of shape variation. The alternative approach (i.e., computing this statistic on both the symmetric and the asymmetric component) would produce similar values for limited amounts asymmetry (which is what we expect in our dataset and at its phylogenetic scale).

**Test of bias**

To test for the presence of bias we conducted a resampling-based test of the null hypothesis of no difference in means across repeated measures. It has been noticed (Fruciano 2016) that most, if not all, the investigations of bias in geometric morphometric data across conditions carried out to date (most commonly comparing fresh and preserved specimens) use an inappropriate resampling scheme in hypothesis testing. Indeed, these use permutation tests – most commonly in MorphoJ – designed to test for differences in mean shape between two independent groups using Procrustes distance as test statistic. These test randomly permute observations across the two groups and these resampling schemes reflect the exchangeability of observation across the two groups under the null hypothesis of no differences between groups (i.e., any observation of one group is exchangeable with any observation in the other, under the null hypothesis). However, in the case of repeated measures, the same individuals are measured in a treatment/control fashion so, under the null hypothesis of no treatment effect, each observation of one treatment is only exchangeable with the observation of the same individual in the other treatment. For this reason, here – using a custom MATLAB script – we test the null hypothesis of no difference between conditions (i.e., surfaces obtained on the same individuals using different devices, surfaces obtained using the same device but digitized by different operators) using permutations within subjects. Incidentally, this approach also allows a more realistic inference of the existence of bias across repeated measurements. Indeed, using inappropriately a test designed for independent groups in most cases results in a failure to reject the null hypothesis (i.e., a non-significant p-value) as it is very unlikely that the bias is so large to overcome differences among individuals. In practice, using an inappropriate resampling scheme results in a reassuring – but misguided – non-significant difference between treatments.

**Measurement error using automated approaches**

Here, to analyse the extent to which automated approaches working on surfaces are affected by variation due to the device used for acquiring surfaces, we use a recently proposed approach (Pomidor et al. 2016), as implemented in the software GPSA. This method superimposes surfaces by establishing a correspondence between points on one surface and their nearest neighbour on the other surface and then minimizing a cost function which is a reflection of the distance between corresponding points. The software also produces ordinations of the observations after superimposition obtained through principal coordinate analysis of distances between observations.

The starting material for this set of analyses was the same set of surface scans from three different devices we used for the analysis of measurement error using human digitization. However, the presence of small “holes” in surfaces – which does not constitute a problem when landmarks are digitized by a human operator – could potentially create problems in the analysis (as “holes” would be treated like biologically-relevant features by the algorithm). On the other hand, a “manual” closing of all holes on each surface scan by a human operator in software such as Meshlab would require a very long time, thereby defeating one of the main reasons for using automated approaches. To reach a “happy medium” we, therefore, implemented a semi-automated procedure in Meshlab to close holes which required minimal operator intervention and time. This consisted in applying to all surfaces a set of Meshlab scripts with parameters empirically determined to give good results on a subset of surfaces. Using this set of scripts, we first obtained a new closed surface using Poisson reconstruction (octree depth 12, solver divide 10, 1 sample per node), then we merged the original and the new surface (also applying a filter to remove duplicate faces and removing all faces with an edge longer than 50). Finally, we first decimated the resulting number of faces in half (because merging the original and new surfaces roughly doubles the number of faces) and removed duplicated vertices again. As a final step, to make the GPSA analysis possible with 16Gb of computer RAM, we decimated all the resulting surfaces to 300000 faces (which is in the same order of magnitude of the smallest set of surfaces in our sample, the ones obtained by photogrammetry).

The surfaces thus obtained were subjected to the analysis in GPSA and the principal coordinate scores obtained by the program used as data in analyses of measurement error by performing both Procrustes ANOVA (and computation of repeatability) and the test for bias described for landmark data. We performed these analyses on the full set of principal coordinate scores (68 non-zero principal coordinates) and on a reduced set of principal coordinates (first five principal coordinates) determined using a procedure for dimensionality reduction. Using dimensionality reduction prior to downstream analysis has been suggested by the authors of the method themselves. Here, we use a similar approach to the one used by Pomidor and colleagues, based on a scree plot of variance explained for each dimension. The main difference is that we chose an explicit criterion to define the threshold at which to retain or exclude dimensions. Here, as a criterion, we used a comparison to the expected distribution of variance across subsequent components under a broken stick model (Jackson 1993, Fruciano et al. 2016). The set of lower-rank components in our dataset each explaining more variance than the corresponding components under a broken stick model was retained.

**Phylogenetic inference**

We analysed an alignment of 33767 base pairs, subdivided in 8 nuclear and mitochondrial markers for 57 species (Supplementary Table VI). The analyses were performed in BEAST 1.8.3 (Drummond et al. 2012). All markers were separated in partitions using the following criteria: RNA stem and RNA loops each constituted a partition, all the other markers were further divided partitioning the first two codons from the third. This results in a total of 14 partitions (2 partitions x 6 markers + 1 partition x 2 markers). Each partition was allowed to have a separate substitution model, which was modelled using a gamma distribution with six categories and which included, in the case of third codon positions, the proportion of invariant sites. The Yule tree prior and the uncorrelated relaxed clock (Drummond et al. 2006) were used for dating, based on four calibrations based on fossils (Supplementary Table VII; (Prideaux and Warburton 2010, Black et al. 2012, Phillips et al. 2013, Travouillon et al. 2014)). Monophyly was not enforced in ingroup calibrated nodes. Two independent runs of 20 million generations, sampled every 2000 generations, were used and convergence was checked by examination of the traces and comparison of median node ages obtained in each run. A phylogenetic tree (Supplementary Fig. 4) with generally high support at nodes was obtained using TreeAnnotator (median dates, 20% used as burn-in) on the distribution of trees of the first run. This tree was used as starting tree for phylogenetic comparative analyses using a single tree. For phylogenetic comparative analyses incorporating phylogenetic information, the first 20% of trees was excluded as burn-in from the posterior distribution of trees of each run and both runs combined for further analyses. We stress that the aim of this analysis was to obtain a distribution of trees with reasonable levels of variation in topology and branch lengths.

**Phylogenetic signal**

All the analyses were run in R (R Core Team 2016).

For the analyses using a single tree, the published code (Adams 2014) for the computation of K_MULT_ was used, pruning the tree as needed. In particular, in addition to the most taxon-rich dataset (Fig. 2), separate analyses were performed for the following subsets: (1) members of the genera *Dendrolagus*, *Petrogale* and *Thylogale* (Clade A), (2) members of the genera *Macropus*, *Wallabia*, *Setonix* and *Onychogalea* (Clade B), (3) members of the genus *Macropus*, (4) members of the genus *Petrogale*. For each of these datasets and subsets, K_MULT_ was computed on each unique combination of device, operator and landmark set (for a total of 3 devices x 2 operators x 2 landmark sets = 12 unique combinations). As for the other analyses, separate Procrustes fits were computed for each subset, so to minimize interspecific distances. We have also extended this approach to random subsets of taxa (5, 10, 15 taxa), computing for each random subset phylogenetic diversity and the coefficient of variation in K_MULT_ across device/operator combinations.

To incorporate phylogenetic uncertainty, we also computed K_MULT_ on all the datasets and subsets mentioned above for each tree of the posterior distribution (excluding burn-in), thus obtaining distributions of K_MULT_, rather than unique values. Due to the high number of computations, we also implemented the computation of K_MULT_ in parallel, using multiple processors. We provide the R code for the implementation we used hoping that this can stimulate the computation of this (and other) statistic on distribution of trees, rather than on single “best” trees (Supplementary Material 1).

To quantify the relative contribution of variation in tree, device and operator to variation in K_MULT_, we used an approach based on an analysis of variance (ANOVA) using the value of K_MULT_ obtained as described above as dependent variable and using two predictors: tree as a fixed factor and device nested within tree (the residual will account for variation between operators, of course). The proportion of variance accounted by each term was obtained by dividing the sum of squares for that term by the total sum of squares. However, given the large number of trees in our distributions, using a normal ANOVA would have been computationally prohibitive (because of the large number of levels in the factor). To overcome this limitation, we used a resampling approach. We randomly sampled (without replacement) 1000 trees from the distribution of trees and performed the ANOVA described above only on the values of K_MULT_ obtained using those trees (across all combinations of device and operators). We repeated this for 1000 times so to obtain a distribution of the various ANOVA values and computed means. It is important to notice that the amount of trees used in the procedure above is of paramount importance as the variation produced by the other factors remains approximately constant (as these are not changed) whereas the variation accounted for by the tree term will increase at increasing number of trees. At the same time, increasing the number of trees makes the computation growingly demanding up to the point of being unfeasible with very large number of trees. We considered 1000 trees as a good approximation as in preliminary runs we obtained very similar values of proportion of variance for the various terms using 100 and 1000 trees.

Adams, D. C. 2014. A Generalized K Statistic for Estimating Phylogenetic Signal from Shape and Other High-Dimensional Multivariate Data. Systematic Biology **63**:685-697.

Black, K. H., M. Archer, S. J. Hand, and H. Godthelp. 2012. The Rise of Australian Marsupials: A Synopsis of Biostratigraphic, Phylogenetic, Palaeoecologic and Palaeobiogeographic Understanding. Pages 983-1078 *in* J. A. Talent, editor. Earth and Life: Global Biodiversity, Extinction Intervals and Biogeographic Perturbations Through Time. Springer Netherlands, Dordrecht.

Bookstein, F. L. 1991. Morphometric Tools for Landmark Data.

Cignoni, P., M. Corsini, and G. Ranzuglia. 2008. Meshlab: an open-source 3d mesh processing system. Ercim news **73**:6.

Drummond, A. J., S. Y. W. Ho, M. J. Phillips, and A. Rambaut. 2006. Relaxed Phylogenetics and Dating with Confidence. PLoS Biology **4**:e88.

Drummond, A. J., M. A. Suchard, D. Xie, and A. Rambaut. 2012. Bayesian Phylogenetics with BEAUti and the BEAST 1.7. Molecular Biology and Evolution **29**:1969-1973.

Fruciano, C. 2016. Measurement error in geometric morphometrics. Development Genes and Evolution **226**:139-158.

Fruciano, C., P. Franchini, F. Raffini, S. Fan, and A. Meyer. 2016. Are sympatrically speciating Midas cichlid fish special? Patterns of morphological and genetic variation in the closely related species Archocentrus centrarchus. Ecology and Evolution **6**:4102-4114.

Jackson, D. A. 1993. Stopping rules in principal components analysis: a comparison of heuristical and statistical approaches. Ecology:2204-2214.

Klingenberg, C. P. 2011. MorphoJ: an integrated software package for geometric morphometrics. Mol Ecol Resour **11**:353-357.

Klingenberg, C. P., M. Barluenga, and A. Meyer. 2002. Shape analysis of symmetric structures: quantifying variation among individuals and asymmetry. Evolution **56**:1909-1920.

Klingenberg, C. P., and G. S. McIntyre. 1998. Geometric morphometrics of developmental instability: analyzing patterns of fluctuating asymmetry with Procrustes methods. Evolution:1363-1375.

Milne, N., and P. O'Higgins. 2002. Inter-specific variation in Macropus crania: form, function and phylogeny. Journal of Zoology **256**:523-535.

Phillips, M. J., D. Haouchar, R. C. Pratt, G. C. Gibb, and M. Bunce. 2013. Inferring Kangaroo Phylogeny from Incongruent Nuclear and Mitochondrial Genes. PLoS ONE **8**:e57745.

Pomidor, B. J., J. Makedonska, and D. E. Slice. 2016. A Landmark-Free Method for Three-Dimensional Shape Analysis. PLoS ONE **11**:e0150368.

Prideaux, G. J., and N. M. Warburton. 2010. An osteology-based appraisal of the phylogeny and evolution of kangaroos and wallabies (Macropodidae: Marsupialia). Zoological Journal of the Linnean Society **159**:954-987.

R Core Team. 2016. A language and environment for statistical computing.

Schlager, S. 2016. Morpho: Calculations and Visualisations Related to Geometric Morphometrics.

Travouillon, K., B. Cooke, M. Archer, and S. Hand. 2014. Revision of basal macropodids from the Riversleigh World Heritage Area with descriptions of new material of Ganguroo bilamina Cooke, 1997 and a new species. Palaeontologia Electronica **17**:1-34.

Wickham, H. 2009. ggplot2: elegant graphics for data analysis. Springer Science & Business Media.
