# Supplementary material for: Sharing is caring? Measurement error and the issues arising from combining 3D morphometric datasets
Source: Ecol Evol. 2017 Jul 31;7(17):7034–46. doi: 10.1002/ece3.3256 (PMC5587461; doi:10.1002/ece3.3256)
Supplement: Supplementary file 3 [file ECE3-7-7034-s003.pdf]

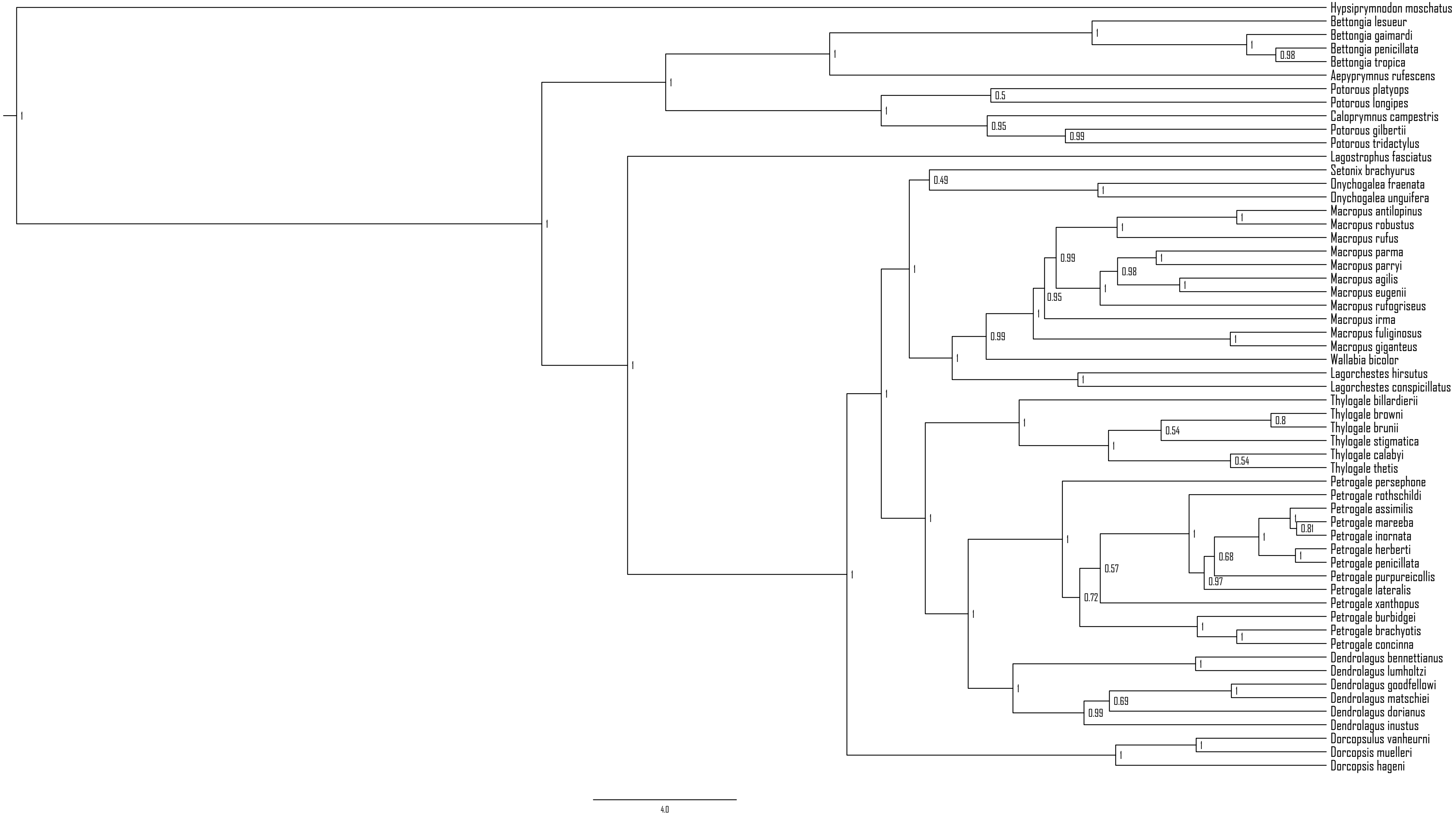
Hypsiprymnodon moschatus
Bettongia lesueur
Bettongia gaimardi
Bettongia penicillata
Bettongia tropica
Aepyprymnus rufescens
Potorous platyops
Potorous longipes
Caloprymnus campestris
Potorous gilbertii
Potorous tridactylus
Lagostrophus fasciatus
Setonix brachyurus
Onychogalea fraenata
Onychogalea unguifera
Macropus antilopinus
Macropus robustus
Macropus rufus
Macropus parma
Macropus parryi
Macropus agilis
Macropus eugenii
Macropus rufogriseus
Macropus irma
Macropus fuliginosus
Macropus giganteus
Wallabia bicolor
Lagorchestes hirsutus
Lagorchestes conspicillatus
Thylogale billardierii
Thylogale browni
Thylogale brunii
Thylogale stigmatica
Thylogale calabyi
Thylogale thetis
Petrogale persephone
Petrogale rothschildi
Petrogale assimilis
Petrogale mareeba
Petrogale inornata
Petrogale herberti
Petrogale penicillata
Petrogale purpureicollis
Petrogale lateralis
Petrogale xanthopus
Petrogale burbidgei
Petrogale brachyotis
Petrogale concinna
Dendrolagus bennettianus
Dendrolagus lumholtzi
Dendrolagus goodfellowi
Dendrolagus matschiei
Dendrolagus dorianus
Dendrolagus inustus
Dorcopsulus vanheurni
Dorcopsis muelleri
Dorcopsis hageni
4.0
